# Neonatal Septic Shoulder Joint Masquerading as Brachial Plexus Palsy: A Case Report and Review of the Literature

**DOI:** 10.1155/crpe/7517956

**Published:** 2025-06-20

**Authors:** Adam Townson, Calver Pang, Lambrini Theocharidou, Sam Bostock, Charles Yuen Yung Loh

**Affiliations:** ^1^Department of Plastic and Reconstructive Surgery, Addenbrooke's Hospital, Cambridge University Hospitals NHS Foundation Trust, Cambridge, UK; ^2^Department of Surgical Biotechnology, Division of Surgery & Interventional Science, Faculty of Medical Sciences, University College London, London, UK

**Keywords:** brachial plexus injury, C5/6 palsy, Erb's palsy, septic arthritis

## Abstract

**Case:** A 2-week-old girl presented to the hospital with a 1-day history of decreased spontaneous movements of her left arm at the shoulder and elbow. There was no history of trauma, and she was otherwise well. Clinically, there was evidence of a C5/6 palsy. MRI of the left shoulder demonstrated an effusion and synovial thickening at the left glenohumeral joint. She was managed with a surgical washout of the joint and intravenous antibiotics. At a 7-month follow-up, she demonstrated a symmetrical range of movement in both shoulders with no signs of acute or chronic infection on X-ray.

**Conclusion:** Septic arthritis of the glenohumeral joint presents insidiously in neonates and infants without typical signs of infection. Neonates and infants presenting with suspected brachial plexus palsy without a convincing history of trauma should raise suspicion of underlying joint infection and be investigated accordingly with blood tests and a low threshold for imaging of the shoulder to facilitate early diagnosis and management.

## 1. Introduction

Brachial plexus palsy (BPP) describes injury to the plexus of nerves that provide motor and sensory input to the upper limb. BPP is a collective term for different palsies which implicate different nerves of the brachial plexus, with the characteristics of each palsy dependent on the specific nerves of the brachial plexus that are implicated. There are various recognised causes of BPP, both congenital and postnatal in origin.

The aetiology of BPP is diverse, with traumatic birth injury as the most common cause [1]. It can arise due to lateral traction of the foetal head during a difficult delivery, in the setting of shoulder dystocia, for example. The traction causes excessive stretching of the nerve bundles, damaging them. However, there is evidence that even when traction is applied properly, BPP can still occur, and it is postulated that the forces generated by uterine contractions during birth are sufficient to generate excessive strain and cause injury to the plexus of nerves. Other causes of BPP include familial congenital BPP, congenital varicella zoster syndrome, aberrant uterine anatomy and malformations, clavicle fracture and other rarer causes such as tumours arising from or invading the brachial plexus, exostosis of the first rib and Kaiser Wilhelm syndrome [2].

Importantly, infection also represents a recognised, albeit rare, cause of BPP in neonates and infants. Both infection of the shoulder joint and osteomyelitis of the humerus, or a combination of the two, have been reported to cause brachial plexopathy in neonates and infants [3–5]. However, typical features of infection are seldom present. Therefore, establishing bone and joint infection as the underlying cause of brachial plexopathy in these neonates and infants is particularly challenging and requires a high index of clinical suspicion. Diagnosis is commonly delayed due to its rarity and insidious nature [5].

In this report, we discuss the case of a 2-week-old girl with a presentation of BPP secondary to septic arthritis of the glenohumeral joint.

## 2. Case Report

A 2-week-old girl presented to the hospital with a 1-day history of decreased spontaneous movements of her left arm at the shoulder and elbow, with otherwise normal movements in the upper limbs prior to this. There was no history of trauma, and she was otherwise clinically well, without evidence of fever or systemic illness.

She had an uncomplicated gestation and was born by an atraumatic vaginal delivery. Birth weight was 2820 g. She had three older siblings who were all healthy.

**Figure 1:**
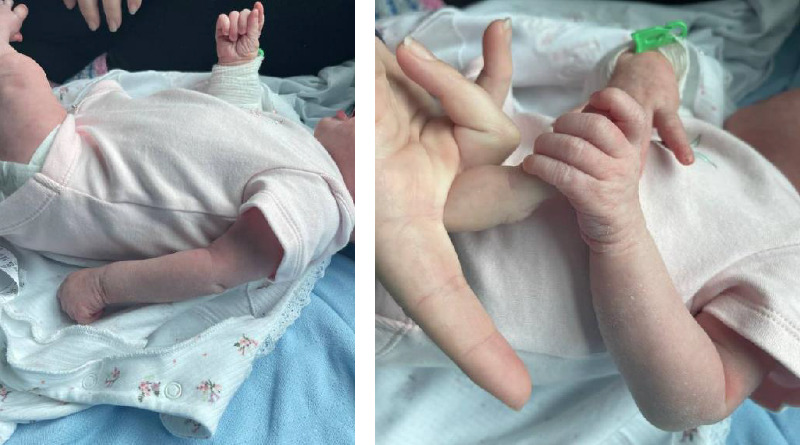
The left waiter's tip suggestive of Erb's palsy (a) and good grip composite motion of fingers and thumb (b).

On examination, she was apyrexial and other vital signs were within normal parameters. She was settled without any obvious signs of pain or distress. On examination, the left upper limb was held in ‘Waiter's tip' resting position (Figure 1(a)). There was no appreciable swelling or skin changes throughout. There was no active movement at the shoulder or elbow, indicative of a C5/6 palsy. Passive movements at these joints did not elicit obvious signs of pain. She was able to actively flex and extend the wrist and had good grip composite motion of the fingers and thumb (Figure 1(b)). Overall, the exam findings were in keeping with Erb's palsy.

X-ray imaging of the shoulder on admission was normal. Blood tests on admission demonstrated a normal white cell count (13.6 × 10^9^/L) and normal liver and renal function. A C-reactive protein (CRP) was not performed on admission due to inadequate blood volume obtained. The underlying infection was not suspected. A magnetic resonance imaging (MRI) scan of the brain was undertaken due to concerns of perinatal stroke and was normal.

**Figure 2:**
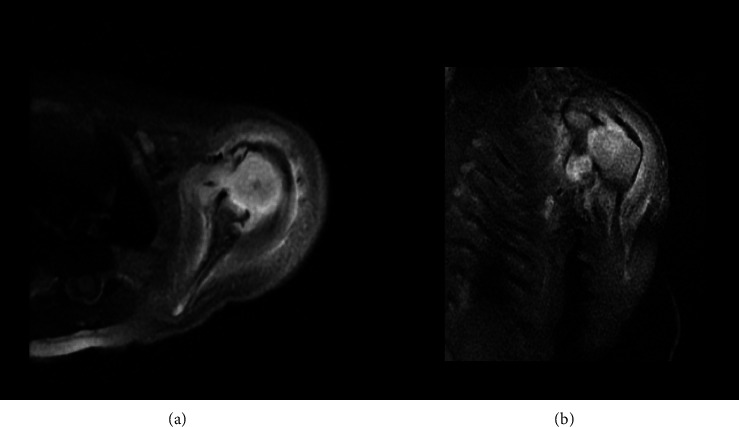
Axial (a) and coronal (b) sections of MRI (*T2-weighted*) images demonstrating left glenohumeral joint effusion and synovial thickening suggestive of synovitis, in keeping with septic arthritis.

An MRI scan of the cervical spine and left shoulder was performed on Day 4 of admission. It demonstrated an effusion and synovial thickening at the left glenohumeral joint, in keeping with florid synovitis (Figure 2). It also demonstrated multiple asymmetrically enlarged left axillary lymph nodes. However, there was no evidence of osteomyelitis, periosteal collection or brachial plexus injury. No spinal pathology was demonstrated. Overall, the images were highly concerning for left glenohumeral joint septic arthritis.

She underwent a washout of the glenohumeral joint in the theatre. Intraoperatively, 1.5 mL of frank pus was aspirated from the joint. Cultures from the aspirate and blood grew *Streptococcus agalactiae.* She was also treated with intravenous antibiotics according to local microbiology advice.

Following the washout of the shoulder joint in theatre, she started to spontaneously move the left arm at the shoulder and elbow joints after a couple of days. She continued to improve and was able to be discharged home and completed a 6-week course of intravenous antibiotics.

At the first follow-up visit, 6 weeks after the initial presentation, the patient was able to move the left shoulder and elbow freely, without any deficit. X-ray of the left shoulder was normal at follow-up. Seven months later, the patient had a symmetrical range of movement in both shoulders with no discernible difference in bulk or length and the X-ray showed good growth and development of the ossific nuclei of the proximal humerus with no signs of acute or chronic infection.

## 3. Discussion

Septic arthritis of the glenohumeral joint can present insidiously in neonates and infants, without typical signs of infection. They may present with reduced movement of the upper limb and suspected BPP. Of the nine previously reported cases [4–12] in the literature, only one patient had a fever or other systemic features of infection on initial presentation, in contrast to the remaining eight patients who presented without any typical signs of infection. This commonly leads to a delay in both presentation to a clinician and a further delay to diagnosis. Of the reported cases, the delay to initial presentation was approximately 1 week, with a further delay of several days to the time of diagnosis. Evidently, establishing bone and joint infection as the underlying cause of brachial plexopathy in a neonate or infant is challenging. However, early diagnosis of a septic joint is essential to enable prompt treatment and minimise long-term damage to the joint or bone, thus improving long-term outcomes. We suggest that neonates and infants who present with BPP without a clear history of trauma must be approached with a high index of suspicion for underlying glenohumeral joint infection, despite its rarity. Early imaging of the shoulder can help determine the presence of joint infection and is therefore recommended. Engaging the wider multidisciplinary team to facilitate MRI scanning is suggested, allowing timely localisation of collections and surgical drainage. It also helps confirm the continuity of the brachial plexus and identify other potential causes of the palsy.

It is often difficult to establish if these patients have a true palsy or what is termed pseudoparalysis, secondary to the effects of pain. Based on history and examination findings alone, it is not always possible to ascertain whether the patient has pseudoparalysis or a true plexopathy. Investigations such as nerve conduction studies (NCS) and electromyography (EMG) can help differentiate between the two, by showing aberrant conduction in nerves, for example. Whilst these investigations were not widely utilised in the existing cases in the literature, there were a few reported cases in which they were performed and clearly showed damage to the nerves rather than pseudoparalysis [4, 11, 12]. While pseudoparalysis may account for some of the patients presenting with BPP, true palsy is certainly possible. The patient in our case did not undergo NCS or EMG as the diagnosis was ascertained and appropriately managed before they could have taken place. Therefore, it is difficult to establish if the patient in our case had pseudoparalysis or a true palsy. In reality, imaging precludes the use of NCS and EMG in the initial workup of these patients, allowing definitive surgical and antibiotic management, meaning that NCS and EMG are not routinely performed so long as the symptoms resolve.

The mechanism by which suppurative arthritis of the glenohumeral joint causes true BPP is not fully elucidated. Given its close anatomical proximity to the brachial plexus, it is possible that inflammation and swelling of the joint capsule results in localised pressure effects on the surrounding neural tissue. Lejman et al. reported four patients with radial nerve palsy secondary to septic arthritis of the glenohumeral joint. Upon dissection, they noted the proximity of the radial nerve passing the distended joint capsule in all four patients, supporting the hypothesis that localised swelling may impinge and compromise the nerves [10, 11].

In conclusion, septic arthritis of the glenohumeral joint presents insidiously in neonates and infants without typical signs of infection. Neonates and infants presenting with suspected BPP without a convincing history of trauma should raise suspicion of underlying joint infection and be investigated accordingly with blood tests and a low threshold for imaging of the shoulder to facilitate early diagnosis and management.

## Data Availability

The data that support the findings of this study are available from the corresponding author upon reasonable request.
